# *NF1* mutations as biomarker of response to immune checkpoint blockades for lung adenocarcinoma patients

**DOI:** 10.1038/s41698-024-00524-x

**Published:** 2024-02-10

**Authors:** Jean-Stéphane Giraud, Anne Jouinot, Eric Pasmant, Camille Tlemsani

**Affiliations:** 1 Institut Cochin, Inserm U1016, CNRS UMR8104, Université Paris Cité, CARPEM, Paris, France; 2 Genetic Department, Hôpital Cochin, AP-HP.Centre-Université Paris Cité, Paris, France; 3 Oncology Department, Hôpital Cochin, AP-HP.Centre-Université Paris Cité, Paris, France

**Keywords:** Health sciences, Lung cancer

## Abstract

Little is known about immune checkpoint inhibitors (ICI) response of *NF1*-mutated lung adenocarcinomas. 341/4,181 (8.2%) TCGA lung adenocarcinomas samples have a somatic *NF1* mutation. *NF1*-mutated tumors have higher TMB (*p* < 0.0001), higher expression of immune genes (“hot phenotype”) and higher CD8 + T cell (*p* = 0.03) and macrophage (*p* = 0.02) infiltrations compared to *NF1* wild-type tumors. *NF1* mutation status appears as a candidate predictive biomarker for ICI response in lung adenocarcinoma patients.

## Introduction

Lung cancer is the leading cause of cancer-related death worldwide^1^, with adenocarcinoma representing the main histological subtype. Immune checkpoint inhibitors (ICI) could be offered to stage IV lung adenocarcinoma patients according to the tumor genotype, PD-L1 expression, patient’s performance status and comorbidities, with a substantial improvement. Several biomarkers have been described to predict immunotherapy response. However, benefits of ICI are only seen in ~15% of cancer patients and there is no strong validated predictor of response used in clinical practice. Identification and development of predictive biomarkers of ICI response are critical.

Somatic mutations in the *NF1* tumor suppressor gene are found in 5% to 15% of lung adenocarcinoma^2^. *NF1* encodes neurofibromin an inhibitor of the RAS/MAPK and PI3K/AKT/mTOR pathways. Several studies have shown that *NF1*-mutated lung adenocarcinoma is a distinct clinical and molecular subtype^3,4^. ICI sensitivity signals have been reported in *NF1*-mutated tumors, mainly in melanoma^5,6^. Only few published clinical data—limited to two case reports^7,8^—showed ICI sensitivity in *NF1*-mutated lung cancer. The imputability of *NF1* mutation in ICI response was not demonstrated in these reports.

We sought to investigate whether *NF1* could be a predictive biomarker of response to ICI in a large cohort of lung adenocarcinoma.

**Fig. 1:**
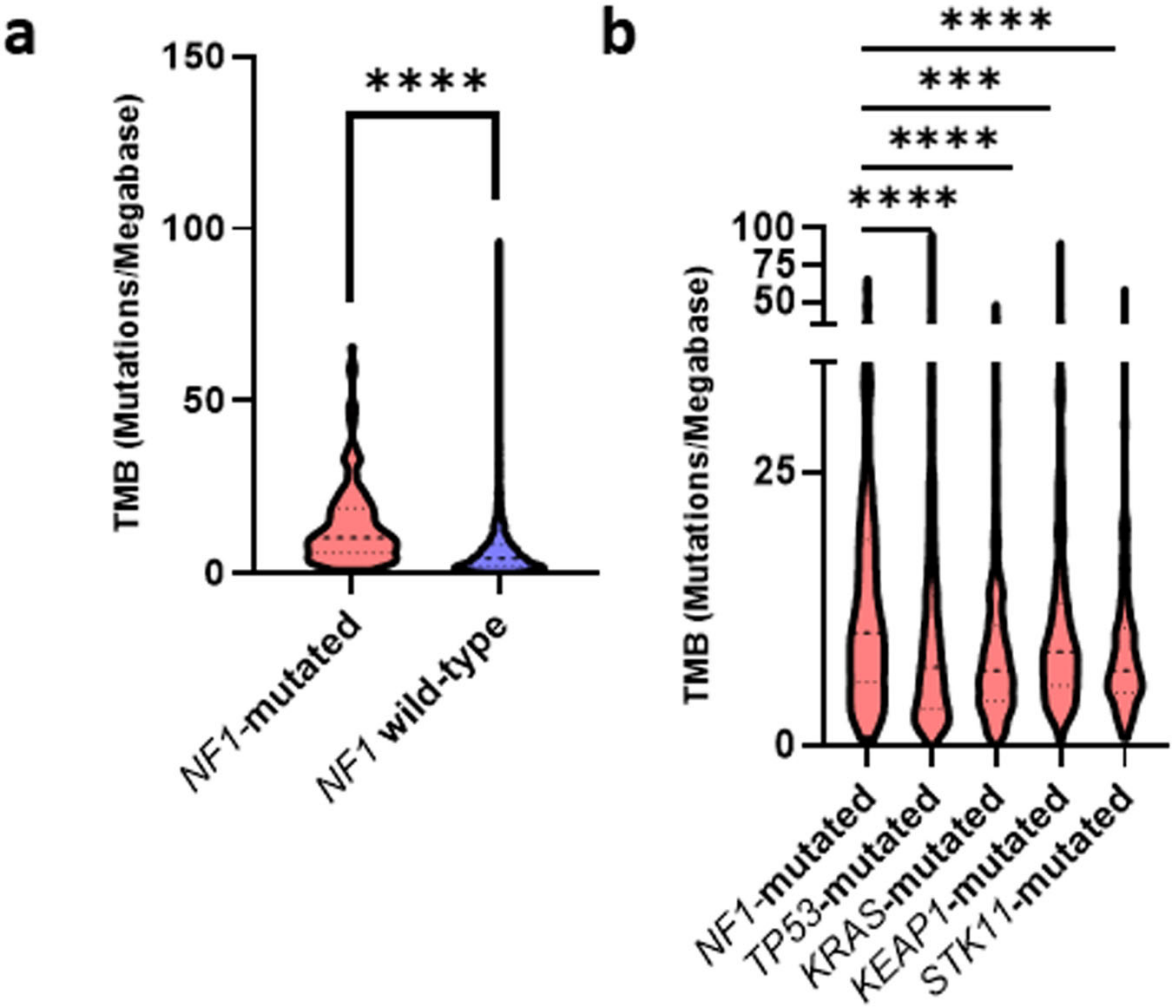
TMB in lung adenocarcinoma according to mutation status. **a** TMB in *NF1* mutated lung adenocarcinomas (*N* = 341 tumors) versus NF1 WT lung adenocarcinomas (*N* = 3840 tumors) showed a statistically significant difference with a mean TMB at 14.1 mut/Mb in *NF1-*mutated tumors *vs* 6.5 mut/Mb in *NF1* WT tumors (*p* < 0.0001). **b** *NF1-*mutated lung adenocarcinomas showed a higher mean TMB *vs TP53*-, *KRAS*-, *KEAP1*-, and *STK11*-mutated lung adenocarcinomas. Numbers of *TP53*, *KRAS*, *KEAP1* and *STK11* co-mutations for the 341 *NF1*-mutated samples are available in Supplementary Table 1. ***P ≤ 0.001 and ****P ≤ 0.0001.

In 14 TCGA cohorts, 341 out of 4181 lung adenocarcinoma patients (8.2%) presented a *NF1* mutation. TMB was significantly higher in *NF1*-mutated *versus NF1* wild-type (WT) lung adenocarcinoma (*p* < 0.0001) with a mean TMB at 14.1 mut/Mb [0.7–65.7] in *NF1*-mutated tumors versus 6.5 mut/Mb [0.0–96.5] in *NF1* WT tumors (Fig. 1, Supplementary Fig. 1). *NF1*-mutated tumors also showed a higher mean TMB (14.1 mutations/Mb) than *TP53-*, *KRAS-*, *KEAP1-*, and *STK11-*mutated tumors (mean TMBs at 10.5, 8.6, 11.2, and 8.8 respectively) (Fig. 1 and Supplementary Table 1). The factors significantly associated with an elevated TMB according to a multivariate analysis are: smoking status (*p* = 0.018) and mutational status of *NF1* (*p* = 0.0183), *TP53* (*p* < 0.0001) and *KEAP1* (*p* = 0.0101) (Table 1).

**Table 1:** Univariate and multivariate logistic regression analysis of factors associated with a high TMB

Variable	Samples with low TMB (<10 Mut/Mb)	Samples with high TMB (≥10 Mut/Mb)	Univariate analysis			Multivariate analysis		
	*n* (%)	*n* (%)	*p*-value	OR	95CI	*p*-value	OR	95CI
***NF1*** **status**			<0.0001	4.441	3.538–5.575	0.0183	2.208	1.145–4.293
Mutated	169 (5%)	172 (19%)						
Wild-type	3124 (95%)	716 (81%)						
***KRAS*** **status**			<0.0001	1.789	1.528–2.094	0.1259	1.451	0.9001–2.341
Mutated	818 (25%)	330 (37%)						
Wild-type	2475 (75%)	558 (63%)						
***TP53*** **status**			<0.0001	5.766	4.866–6.857	<0.0001	7.412	4.522–12.57
Mutated	1206 (37%)	683 (77%)						
Wild-type	2087 (63%)	205 (23%)						
***STK11*** **status**			<0.0001	1.608	1.319–1.953	0.2954	1.352	0.7660–2.382
Mutated	420 (13%)	169 (19%)						
Wild-type	2873 (87%)	719 (81%)						
***KEAP1*** **status**			<0.0001	2.520	2.067–3.066	0.0101	2.118	1.196–3.763
Mutated	323 (10%)	191 (22%)						
Wild-type	2970 (90%)	697 (78%)						
**Smoking status**			<0.0001	6.148	4.554–8.439	0.0018	2.438	1.413–4.341
≥ 20 pack-years	576 (18%)	323 (36%)						
< 20 pack-years	603 (18%)	55 (6%)						
Unknown	2114 (64%)	510 (58%)						
**Age**			0.025	0.7568	0.632– 0.907	0.4776	0.8530	0.550–1.325
High ≥ 64 years old	1140 (35%)	308 (35%)						
Low < 64 years old	874 (27%)	312 (35%)						
Unknown	1279 (39%)	268 (30%)						
**Gender**			0.6521	0.9626	0.815–1.135			
Male	927 (28%)	246 (28%)						
Female	2307 (70%)	636 (72%)						
nknown	59 (2%)	6 (0%)						

In univariate analysis, the age (cut-off: mean), smoking status (cut-off: 20 pack-years), and the mutational status of *NF1*, *KRAS*, *TP53*, *KEAP1* and *STK11* are factors associated with a high TMB. The factors significantly associated with an elevated TMB according to the multivariate analysis are: smoking status (*p* = 0.018) and mutational status of *NF1* (*p* = 0.0183), *TP53* (*p* < 0.0001) and *KEAP1* (*p* = 0.0101). 95CI: 95% confidence interval.

*TMB* Tumor Mutational Burden (mut/Mb), *OR* Odds ratio.

**Fig. 2:**
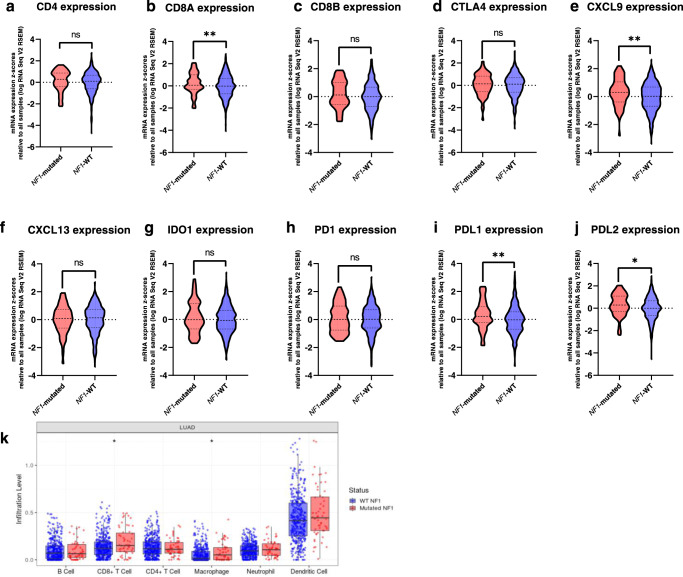
*NF1*-mutated lung adenocarcinoma immune infiltrate profiles. **a**–**j** show the mRNA expression levels of nine genes implicated in inflammation and immune checkpoint inhibitors response, in *NF1*-mutated (*n* = 66) and wild-type (*n* = 620) lung adenocarcinomas*: CD4* (**a**), *CD8A* (**b**), *CD8B* (**c**), *CTLA4* (**d**), *CXCL9* (**e**), *CXCL13* (**f**), *IDO1* (**g**), *PD1* (**h**), *PDL1* (**i**), and *PDL2* (**j**). Each plot represents the mRNA level of key immune genes (*y* axis) in *NF1*-mutated (in red) and WT (in blue) tumors (*x* axis). A significant increase in *CD8A, PDL1, CXCL9*, and *PDL2* expression is observed in *NF1*-mutated vs WT tumors. **k** shows the immune infiltrate abundances (*y* axis) for B cells, CD4^+^ T cells, CD8^+^ T cells, dendritic cells, macrophages, and neutrophils (*x* axis) in *NF1-*mutated (in red) and WT (in blue) tumors. A significant CD8^+^ and macrophage infiltration increase is observed in *NF1*-mutated vs WT tumors. WT: wild-type for *NF1*. *p ≤ 0.05; **P ≤ 0.01; ***P ≤ 0.001 and ****P ≤ 0.0001.

We compared the mRNA levels of nine immune genes in *NF1-*mutated (*N* = 66; 9.6%) *vs NF1* WT (*N* = 620; 90.4%) lung adenocarcinomas (Fig. 2). We observed a significantly higher expression of CXCL9 (*p* = 0.008), PD-L1 (*p* = 0.010), PD-L2 (*p* = 0.011), and CD8A (*p* = 0.006) in *NF1-*mutated samples vs *NF1* WT samples. Lung adenocarcinoma samples with a TMB ≥ 10 mut/Mb also had a higher expression of these same genes compared to samples with a TMB < 10 mut/Mb. *TP53*-mutated samples had a significantly higher expression of all investigated immune markers than *TP53* WT samples. Conversely, *KRAS*-mutated samples showed no increase in the expression of immune genes compared to *KRAS* WT tumors. *KEAP1* and *STK11-*mutated tumors showed a significant decrease in the expression of the nine immune genes vs WT tumors (Supplementary Table 2).

Using the 542 lung adenocarcinoma samples available in TIMER database, we estimated the abundance of six different cell types: B cells, CD4^+^ T cells, CD8^+^ T cells, dendritic cells, macrophages, and neutrophils. *NF1* mutations were associated with significantly higher CD8 + T cells (*p* = 0.03) and macrophage (*p* = 0.02) infiltrations in lung adenocarcinoma compared to *NF1* WT tumors (Fig. 2). *TP53* mutations were associated with significantly higher CD8 + T cells (*p* = 0.01), neutrophils (*p* = 0.001), and dendritic cells (*p* = 0.03) infiltrations. Conversely, *KRAS* mutations were associated with significantly lower B cells (*p* = 0.02) and dendritic cells (*p* = 0.04) infiltrations. *STK11* and *KEAP1* mutations were associated with significantly lower infiltrations of all six markers (all *p* < 0.03) (Supplementary Fig. 2).

**Fig. 3:**
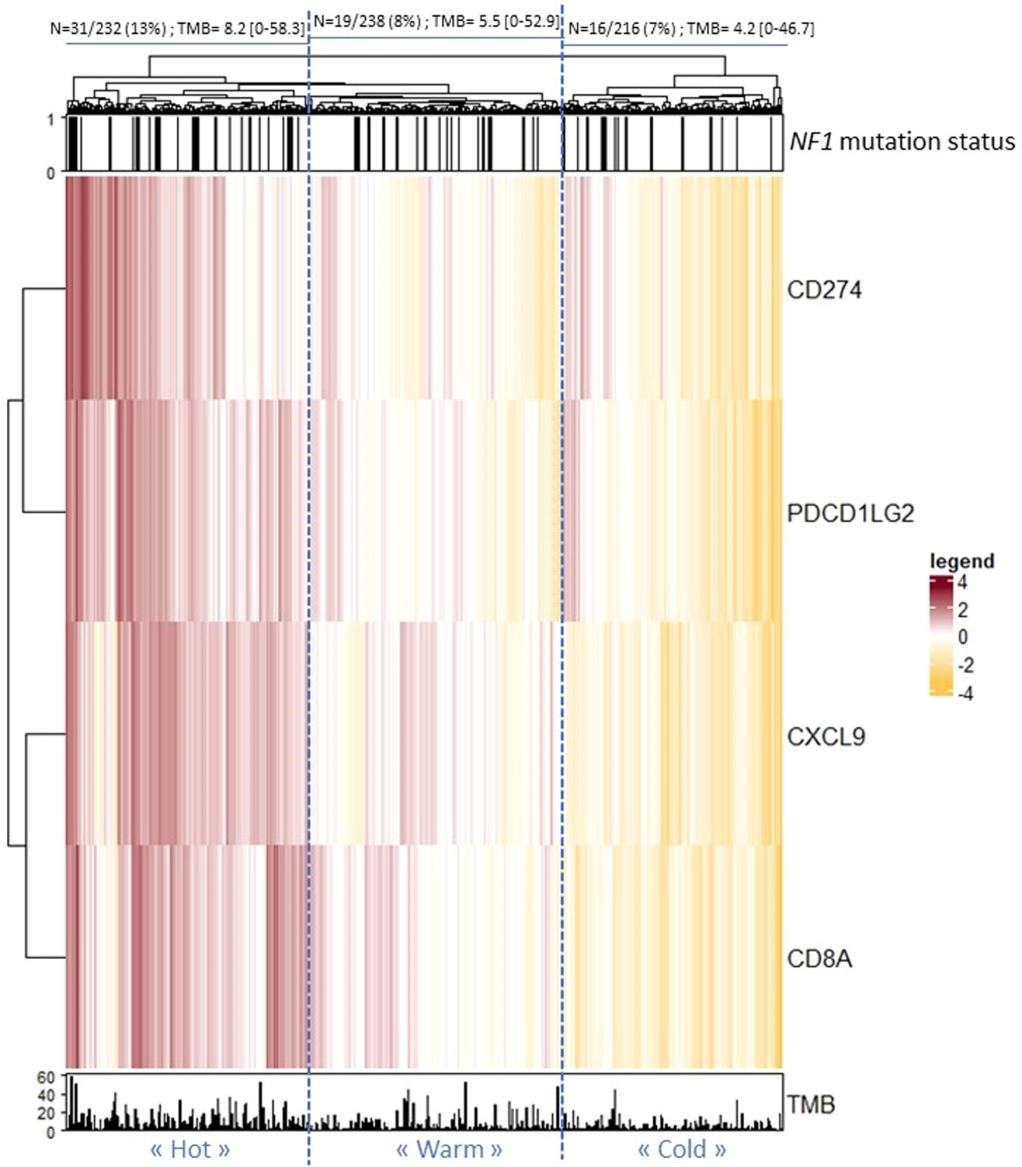
Lung adenocarcinoma clustering according to the mRNA expression of immune genes (*N* = 686). Heatmap shows the expression of PD-L1 (encoded by *CD274*), PD-L2 (encoded by *PDCD1LG2*), CXCL9, and CD8A. *NF1* mutation status (upper) and TMB (lower) are indicated. The number of *NF1* patients and the TMB [mean (95CI)] based on a three-part clustering: “hot”, “warm” and “cold” tumors are indicated. TMB: Tumor Mutational Burden (mut/Mb). 95CI: 95% confidence interval.

A heatmap representing the mRNA expression z-score in 686 lung adenocarcinomas with available expression data (*NF1* mutant: *N* = 66, 9.6%; *NF1* WT: *N* = 620, 90.4%) is shown (Fig. 3). We identified three clusters of tumors according to the level of expression of these immune genes, that we named “hot tumors”, “warm tumors”, and “cold tumors”. A significant enrichment of *NF1*-mutated tumors was identified in “hot tumors”: OR[IC95] = 1.84[1.07, 3.18] (Fisher *p* = 0.02015). No *NF1*-mutated tumors enrichment was identified in “warm” (OR[IC95] = 0.74[0.40, 1.32], Fisher *p* = 0.3416) or in “cold” tumors (OR[IC95] = 0.67[0.35, 1.24], Fisher *p* = 0.2106). TMB was higher in “hot” tumors than in “warm” (*p* = 0.013) and “cold” (*p* < 0.001) tumors. All *NF1*-mutated samples with available expression data had a TMB > 10 mut/Mb.

In a 2023 single-center retrospective study, Wang et al. showed an enhanced proliferation and immune activity of macrophages and NK cells in case of germline *NF1* mutations^9^ in patients with juvenile myelomonocytic leukemia. Data are available for *NF1*-mutated sporadic cancers—which mainly include melanoma. Johnson et al. conducted a study on 65 patients with advanced melanoma treated with ICI. The subgroup of patients with *NF1*-mutated melanomas had a higher TMB and better response rates (74%) than those with *BRAF/NRAS*-mutated and wild-type melanomas^5^. Furthermore, a retrospective multicenter analysis revealed a significantly better median OS (*p* = 0.0154) when receiving first-line immune checkpoint inhibitor treatment for *NF1*-mutated (*n* = 80) than for wild-type (*n* = 432) melanomas^6^. However, there are currently no clinical trials evaluating ICI efficacy that enrolled patients according to a stratified randomization on their *NF1* status^2^. In the context of lung adenocarcinomas, only two case reports showed durable responses to ICI in two patients with sporadic *NF1*-mutated cancers. The first one showed a progression free survival of 95.4 weeks with pembrolizumab^7^. The second reported a stable disease for 8 months in a stage IV lung adenocarcinoma patient treated with pembrolizumab^8^. These observations support the need to explore *NF1* as a predictive biomarker of ICI sensitivity in the context of lung adenocarcinoma.

MSK-IMPACT study showed that response to ICI is associated with a high TMB, whatever tumor histological subtype. In lung cancers, an association was identified between an elevated TMB and durable clinical benefits of ICI^10–12^. Three studies presented at recent congresses confirmed a significantly higher TMB (*P* < 0.001, *p* < 0.0001 and *p* < 0.0001) and a higher expression of PD-L1 expression (*p* < 0.01, *p* = 0.05 and *p* < 0.0001) in *NF1*-mutated versus *NF1*-WT lung cancers^13–15^. Our in silico analysis of 686 samples confirmed these results: *NF1* mutation was associated with a higher TMB (*p* < 0.0001) and a higher expression of key immune genes. We showed a significant enrichment of *NF1*-mutated samples in “hot tumors” (*p* = 0.02) and an increased CD8 + T-cell infiltration (*p* = 0.03).

Given that *NF1*-mutated lung adenocarcinoma is frequently co-mutated (and especially with *TP53*), one cannot exclude an impact of these co-mutations on the TMB and immune infiltration. However, our multivariate analysis shows that *NF1* status is significantly associated (*p* = 0.0183) with high TMB independently of the co-mutation status. *TP53* mutations have been reported to be a biomarker associated with ICI benefit response, in particular in case of *KRAS* co-mutations^10^. A 2017 clinical study aimed to identify novel biomarkers for immune check-point inhibitor responses in 904 patients with lung adenocarcinoma^16^. Patients with *TP53*-mutated lung adenocarcinoma were characterized by a significant increased expression of PD-L1 (*p* < 0.05) and TMB (*p* < 0.001), and a higher tumor infiltration by CD8 + T cells (*p* < 0.05). In 2020, a study of 637 patients with non-small cell lung cancer (NSCLC) confirmed a high TMB (*p* = 0.007) and high PD-L1 expression (*p* < 0.001) in *TP53*-mutated samples^10^. *NF1* mutation status was not investigated in these studies. Here, we confirmed that *TP53* mutations were correlated with higher TMB (*p* < 0.0001) and higher expression of immune markers. Pilar et al. showed that the combination of multiple biomarkers (PD-L1 immunohistochemistry, T-cell infiltration, and TMB) increased performance, compared with the three biomarkers alone^17^. Our results suggest that a mixed score including *NF1* should be considered.

The presence of *NF1* mutations was correlated with high TMB. This sole observation did not allow to determine if *NF1* mutations could be a consequence of genetic instability. The transcriptional and immune analyses were limited to small datasets. Because of this limited size, we could not check whether the subgroup of *NF1*-mutated patients with low TMB expressed markers of immune infiltration. In addition, one of the limitations of in silico analysis is the lack of available clinical data. We did not have sufficient clinical outcomes of patients with *NF1*-mutated lung adenocarcinoma and treated with ICI in these TCGA public databases. We were unable to make a multivariate model to predict the efficacy of ICI. We therefore looked for factors associated with a high TMB, that is correlated with durable clinical benefits of ICI. To study the impact of genes on ICI response, a progression free survival analysis would have been necessary. Additional studies are warranted to specify the biological effect of *NF1* mutations on immune environment in lung adenocarcinoma.

We showed that *NF1*-mutated lung adenocarcinomas are associated with a high TMB, high immune genes expression, and CD8 + T-cells infiltration. To date in clinical practice, besides PD-L1 expression that is actually use to drive the choice of immunotherapy regimens in non-small cell lung cancer that lacks a driver mutation, there is no strong predictive factor of immune checkpoint blockade. We suggest the potential of *NF1* alterations as a novel biomarker for ICI, warranting further investigations alone or in combination with other ICI predictive biomarkers. Our results highlight the need for clinical trials evaluation ICI efficacy specifically dedicated to *NF1*-mutated lung adenocarcinomas with a randomization based on *NF1* status.

## Methods

### Data acquisition

We studied in silico data using 14 publicly available datasets with lung adenocarcinoma samples on the cBioPortal website (The Cancer Genome Atlas [TCGA] data, https://www.cbioportal.org/): MSKMind (Nature Cancer 2022, *n* = 247 samples), Broad (Cell 2012, *n* = 183 samples), CPTAC (Cell 2020, *n* = 110 samples), MSK (2021, *n* = 186 samples), MSK (J Thorac Oncol 2020, *n* = 604 samples), MSK (NPJ Precision Oncology 2021, *n* = 426 samples), MSK (Science 2015, *n* = 35 samples), OncoSG (Nat Genet 2020, *n* = 305 samples), TCGA (Firehose Legacy, *n* = 586 samples), TCGA (Nature 2014, *n* = 230 samples), TCGA (PanCancer Atlas, *n* = 566 samples), TSP (Nature 2008, *n* = 163 samples), NCI (Nature Genetics 2021, *n* = 232 samples) and MSK (Cancer Discov 2017, *n* = 915 samples). After removing non-lung adenocarcinoma samples and duplicates, 4181 lung adenocarcinoma samples were included in the analysis.

### Tumor mutational burden and genetic alterations

We analyzed the presence and type of *NF1* alterations, the level of *NF1* mRNA expression (log2 RNA seq V2 RSEM), and the tumor mutational burden (TMB) defined as the number of mutations per megabase (mut/Mb).

We compared the *NF1*-mutated lung adenocarcinomas profile with *TP53-* and *KRAS*-mutated tumors which are likely associated with ICI response^10,16,18^, and *KEAP1-* and *STK11*-mutated tumors which are likely known to be resistant to ICI^18–21^. For *KRAS*-mutated tumors, we only included tumors with *KRAS* hotspot mutations.

### Tumor immune infiltrates

We determined the tumor immune infiltrates using the mRNA expression level of markers known to be predictive of ICI response^17,22,23^: PD-1, PD-L1, PD-L2, CTLA4, IDO, CXCL9, CXCL13, CD4, and CD8. Only five datasets had mRNA expression data: Firehorse Legacy (*n* = 341 lung adenocarcinoma), PanCancer Atlas (*n* = 196 lung adenocarcinoma), Nature 2014 (*n* = 49 lung adenocarcinoma), Nat Genet 2020 (*n* = 304 lung adenocarcinoma), and Cell 2020 (*n* = 110 lung adenocarcinoma). After removing duplicates and samples without mRNA expression of the tested immune markers, the mRNA expression analysis was performed in 686 samples.

Using TIMER (Tumor Immune Estimation Resource, https://cistrome.shinyapps.io/timer/), a public web server for systematical analysis of immune infiltrates, we estimated the abundance of six immune cell populations including B cells, CD4^+^ T cells, CD8^+^ T cells, dendritic cells, macrophages, and neutrophils in 542 lung adenocarcinoma samples.

### Statistical analyses

Comparisons were performed with Student t-test or Wilcoxon for quantitative variables. ANOVA with false discovery rate correction (two-stage step-up method) was performed for multi-group comparison. Clustering analyses and heatmap visualization were generated using the “ComplexHeatmap” R package. Samples clustering was performed according to the mRNA expression level of significant immune genes. Enrichment score was calculated using the Fisher exact test. We set up univariate and multivariate logistic regression models to determine the factors associated with a high TMB (defined as >10 Mut/Mb). We included as clinical and genomic criteria: age, gender, smoking status (cut-off: 20 pack-years) and the mutational status of *NF1*, *KRAS*, *TP53*, *KEAP1* and *STK11*. All *p*-values were two-sided, and the level of significance was set at *p* < 0.05. Statistical analyses were performed with R statistical software (version 4.2.2) and GraphPad Prism (version 10.0.0).

### Reporting summary

Further information on research design is available in the Nature Research Reporting Summary linked to this article.

### Supplementary information

Supplementary information

Reporting summary

## Data Availability

This study is based on 14 publicly available datasets with lung adenocarcinoma samples on the cBioPortal website (The Cancer Genome Atlas, https://www.cbioportal.org/): MSKMind (Nature Cancer 2022), Broad (Cell 2012), CPTAC (Cell 2020), MSK (2021), MSK (J Thorac Oncol 2020), MSK (NPJ Precision Oncology 2021), MSK (Science 2015), OncoSG (Nat Genet 2020), TCGA (Firehose Legacy), TCGA (Nature 2014), TCGA (PanCancer Atlas), TSP (Nature 2008), NCI (Nature Genetics 2021) and MSK (Cancer Discov 2017).

## Code availability

Statistical analyses were performed with GraphPad Prism (version 10.0.0) using Student t-test, Wilcoxon test, ANOVA with false discovery rate correction (two-stage step-up method), Fisher exact test and univariate and multivariate logistic regression models. Clustering analyses and heatmap visualization were generated with R statistical software (version 4.2.2) using several R packages: “tidyverse”, “ComplexHeatmap” and “colorRamp2”.
