# Supplementary material for: NF1 mutations as biomarker of response to immune checkpoint blockades for lung adenocarcinoma patients
Source: NPJ Precis Oncol. 2024 Feb 10;8:32. doi: 10.1038/s41698-024-00524-x (PMC10858913; doi:10.1038/s41698-024-00524-x)
Supplement: Supplementary file 1 — Supplementary information [file 41698_2024_524_MOESM1_ESM.pdf]

Supplementary Information

Supplementary Table 1. ***NF1*, *TP53*, *KRAS*, *KEAP1*, and *STK11* alterations and TMB details in the 14 lung adenocarcinoma TCGA cohorts (N=4,181 tumor samples)**

	NF1	*TP53*	*KRAS*	*KEAP1*	*STK11*
**Total number of samples with somatic mutation**	**341 (8.2%)**	**1,889 (45.2%)**	**1,148 (27.5%)**	**514 (12.3%)**	**589 (14.1%)**
Number of samples with missense mutations	132	1,241	1,148	394	158
Number of samples with nonsense mutations	80	277	0	43	158
Number of samples with splice mutations	65	151	0	11	92
Number of samples with fusions	5	3	0	0	12
Number of samples with frameshift indels	59	183	0	60	163
Number of samples with indels in frame	0	34	0	6	6
**Co-mutations with *NF1***: n (association: OR[IC95], Fisher p-value)	/	233 (co-occurring: 2.85[2.25, 3.60], p <0.0001)	75 (mutually exclusive: 0.73[0.56, 0.95], p = 0.0223)	59 (co-occurring: 1.56[1.15, 2.10], p = 0.0045)	50 (no association: 1.05[0.77, 1.44], p = 0.7454)
**Tumor Mutational Burden** (TMB, mutations/megabase): mean [min-max] (comparison to *NF1*-samples: adjusted p-value)	**14.1 [0.7-65.7]**	**10.5 [0.0-96.5] (<0.0001)**	**8.6 [0.1-48.3] (<0.0001)**	**11.2 [0.2-90.4] (<0.0001)**	**8.8 [0.7-58.7] (<0.0001)**

Supplemental Table 2. Statistical p-values of the difference in mRNA expression level for genes known to be implicated in inflammation and immune checkpoint inhibitors response for wild-type and mutated lung adenocarcinoma (N=686).

mRNA expression	*NF1*	*TMB*	*TP53*	*KRAS*	*KEAP1*	*STK11*
CD4	No difference between mutated and WT tumors (p=0.1805)	No difference between high and low TMB tumors (p=0.1529)	Higher in *TP53*-mutated tumors (p=0.0230)	Lower in *KRAS*-mutated tumors (p<0.0001)	No difference between mutated and WT tumors (p=0.5018)	Lower in *STK11*-mutated tumors (p<0.0001)
CD8A	Higher in *NF1*-mutated tumors (p=0.0057)	Higher in tumors with a TMB > 10 (p=0.0133)	Higher in *TP53*-mutated tumors (p=0.0043)	No difference between mutated and WT tumors (p=0.1463)	No difference between mutated and WT tumors (p=0.4450)	No difference between mutated and WT tumors (p=0.4062)
CD8B	No difference between mutated and WT tumors (p=0.1433)	No difference between high and low TMB tumors (p=0.0829)	Higher in *TP53*-mutated tumors (p=0.0270)	No difference between mutated and WT tumors (p=0.1127)	No difference between mutated and WT tumors (p=0.2824)	No difference between mutated and WT tumors (p=0.2363)
CD274 = PD-L1	Higher in *NF1*-mutated tumors (p=0.0096)	Higher in tumors with a TMB > 10 (p= 0.0076)	Higher in *TP53*-mutated tumors (p<0.0001)	Lower in *KRAS*-mutated tumors (p=0.0002)	No difference between mutated and WT tumors (p=0.4263)	Lower in *STK11*-mutated tumors p<0.0001)

CTLA4	No difference between mutated and WT tumors (p=0.6136)	No difference between high and low TMB tumors (p=0.5678)	Higher in *TP53*-mutated tumors (p=0.0083)	Lower in *KRAS*-mutated tumors (p=0.0008)	No difference between mutated and WT tumors (p=0.5885)	No difference between mutated and WT tumors (p=0.0948)
CXCL9	Higher in *NF1*-mutated tumors (p=0.0084)	Higher in tumors with a TMB > 10 (p<0.0001)	Higher in *TP53*-mutated tumors (p<0.0001)	No difference between mutated and WT tumors (p=0.8144)	No difference between mutated and WT tumors (p=0.1563)	Lower in *STK11*-mutated tumors (p=0.0449)
CXCL13	No difference between mutated and WT tumors (p=0.9468)	No difference between high and low TMB tumors (p=0.4847)	Higher in *TP53*-mutated tumors (p=0.0352)	No difference between mutated and WT tumors (p=0.7353)	No difference between mutated and WT tumors (p=0.7983)	No difference between mutated and WT tumors (p= 0.4776)
IDO1	No difference between mutated and WT tumors (p=0.2093)	No difference between high and low TMB tumors (p=0.6743)	Higher in *TP53*-mutated tumors (p<0.0001)	Lower in *KRAS*-mutated tumors (p=0.0169)	Lower in *KEAP1*-mutated tumors (p=0.0020)	Lower in *STK11*-mutated tumors (p= 0.0323)
PDCD1 = PD-1	No difference between mutated and WT tumors	No difference between high and low TMB tumors	Higher in *TP53*-mutated tumors (p<0.0001)	No difference between mutated and WT tumors	No difference between mutated and WT tumors	No difference between mutated and WT tumors

	(p=0.3790)	(p=0.0716)		(p=0.0583)	(p=0.4293)	(p=0.1166)
PDCD1LG2 = PD-L2	Higher in *NF1*-mutated tumors (p=0.0115)	Higher in tumors with a TMB > 10 (p=0.0175)	Higher in *TP53*-mutated tumors (p=0.0024)	Lower in *KRAS*-mutated tumors (p=0.0017)	Lower in *KEAP1*-mutated tumors (p=0.0036)	Lower in *STK11*-mutated tumors (p<0.0001)

We calculated the p-value of the difference in mRNA expression between wild-type (WT) and mutated specimen for each gene (*NF1, KEAP1, KRAS, STK11* and *TP53*) and when TMB (Tumor Mutational Burden) was high (>10 mutations/megabase) versus low (<10 mutations/megabase). In red are the significant p-values when the expression is higher in case of mutated tumors and in blue the significant p-values when the expression is lower in case of mutated tumors.

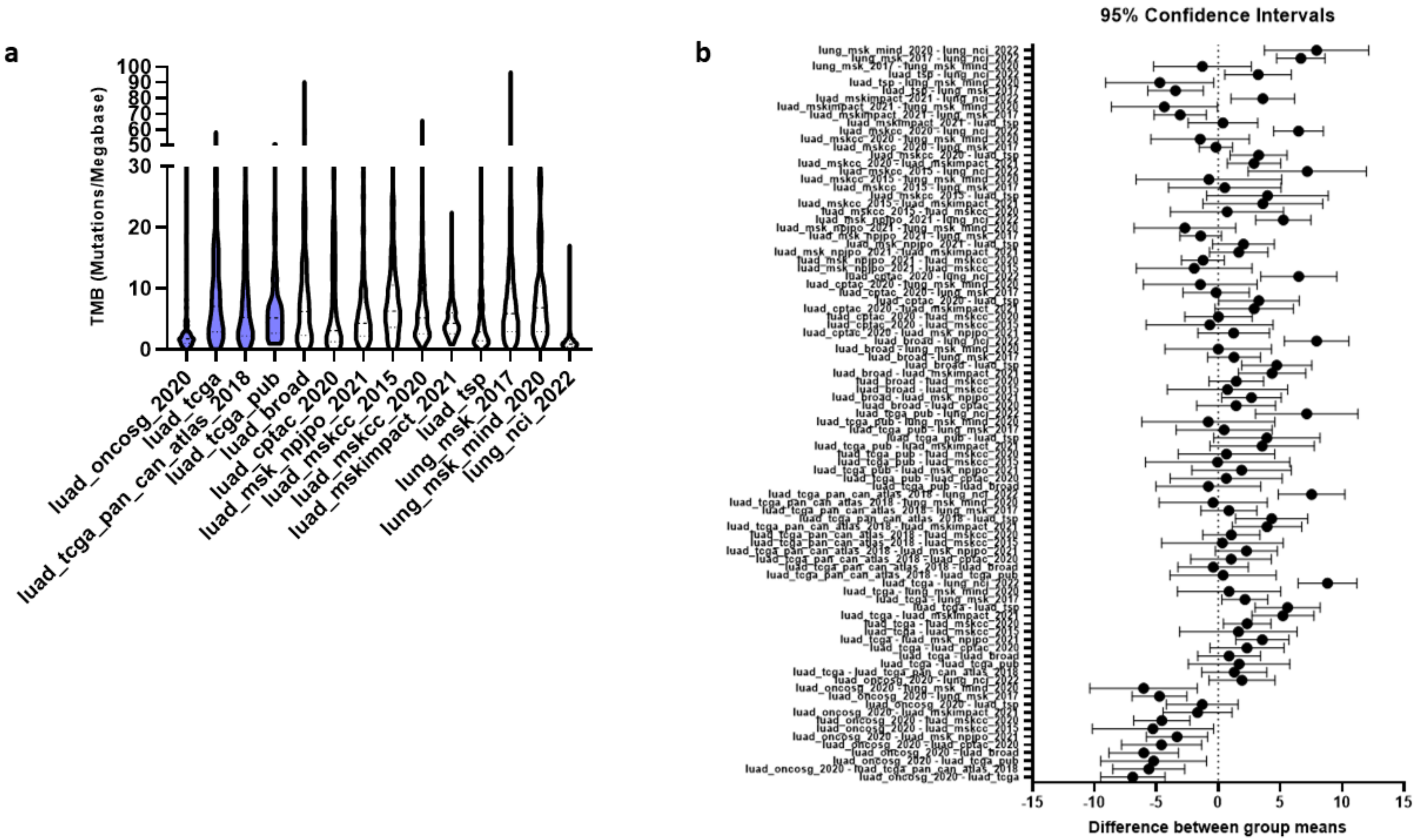

Supplementary Figure 1. TMB distribution amongst studied TCGA datasets. (A) violin plot represents TMB (y axis) for each studied TCGA datasets (x axis). Cohorts colored in purple are those with expression data (used for immune gene expression analysis). (B) plot represents the 95% confidence intervals of the mean difference (x axis) for the cohorts (y axis). They were calculated using a Tukey's multiple comparison test. The TMB distribution appears to be similar between the different cohorts. TMB: Tumor Mutational Burden (mut/Mb).

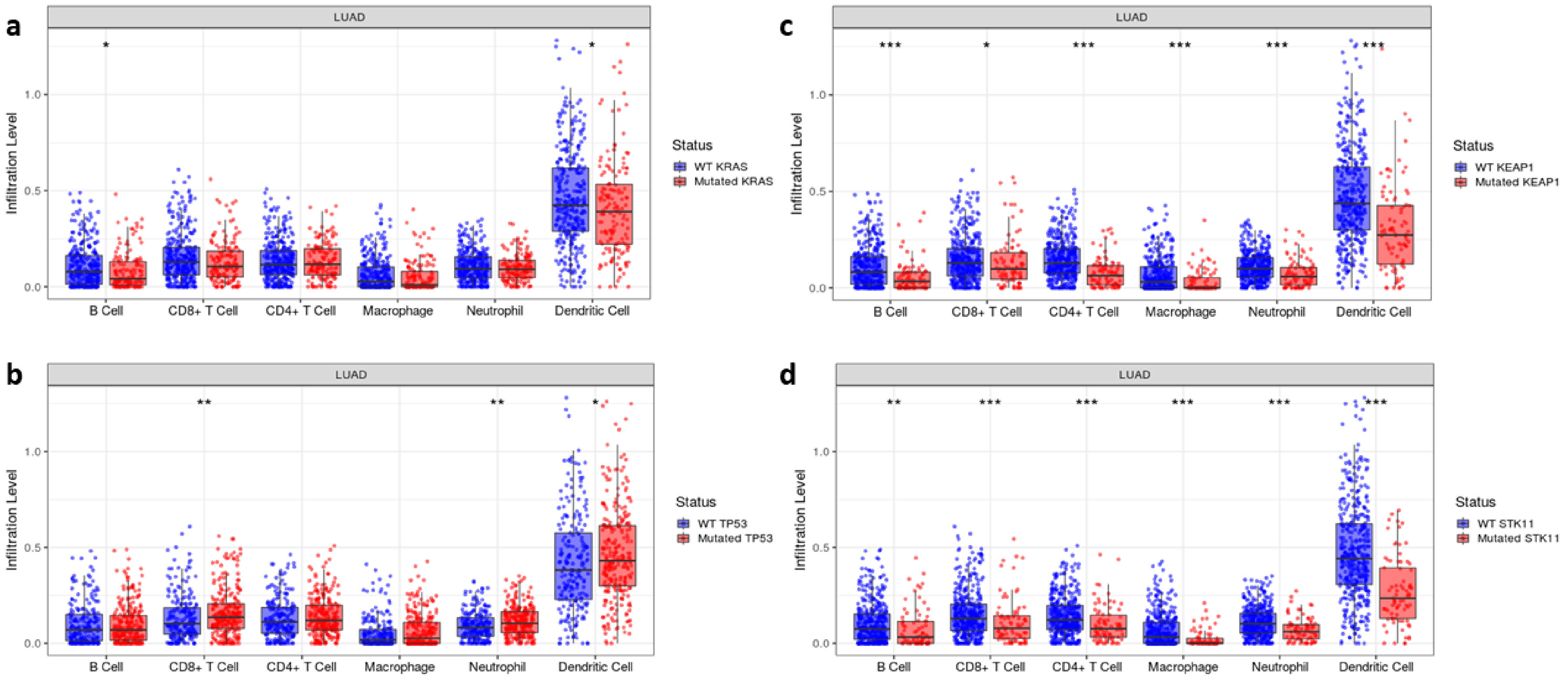

Supplementary Figure 2. Analysis of the lung adenocarcinoma tumor immune infiltrates profile in case of *KRAS, TP53, KEAP1* or *STK11* mutation. (A) to (D) panels show the immune infiltrates abundances depending on *NF1* mutation status in 542 lung adenocarcinoma samples available on TIMER. The plot represents immune infiltration levels (y axis) for B cells, CD4$^+$ T cells, CD8$^+$ T cells, dendritic cells, macrophages and neutrophils (x axis) according to the mutated (in red) and wild-type (in blue) tumors for the following genes: *KRAS* (A), *TP53* (B), *KEAP1* (C) and *STK11* (D). There is a significant CD8+ and denditric cell infiltrations in case of *TP53* mutation compared to *TP53* wild-type tumors. There is a significantly lower infiltrations of B, CD8$^+$, CD4$^+$, macrophage, neutrophil and denditric cells in case of *KEAP1 or STK11* mutation compared to *KEAP1 or STK11* wild-type tumors. WT: wild-type. * for p ≤ 0.05 ; ** for P ≤ 0.01 ; *** for P ≤ 0.001 and **** for P ≤ 0.0001.
